# Biomarkers of Sarcopenia and Sarcopenic Obesity in Renal Transplant Recipients: A Systematic Review and Evidence Quality Assessment

**DOI:** 10.3390/jcm14248943

**Published:** 2025-12-18

**Authors:** Ioanna Soukouli, Thomas Karagkounis, Konstantinos S. Mylonas, Theofanis Kalathas, Kalliopi-Anna Poulia, Alexander Kokkinos, Smaragdi Marinaki

**Affiliations:** 1 Department of Nephrology and Renal Transplantation, Laiko General Hospital, National and Kapodistrian University of Athens, 157 71 Athens, Greece; 2 Surgery Working Group, Society of Junior Doctors, 155 71 Athens, Greece; 3 Department of Surgery, Emory University School of Medicine, Atlanta, GA 30322, USA; 4 Department of Medicine, University of Rochester Medical Center, Rochester, NY 14642, USA; 5 Laboratory of Dietetics and Quality of Life, Agricultural University of Athens, 155 71 Athens, Greece; 6 First Department of Propaedeutic and Internal Medicine, Laiko General Hospital, Athens University Medical School, 155 71 Athens, Greece

**Keywords:** sarcopenia, sarcopenic obesity, kidney transplant, renal transplant, biomarker, myostatin, adiponectin, leptin, BDNF, Insulin like Growth Factor-1

## Abstract

**Background:** Sarcopenia and sarcopenic obesity are increasingly recognized in kidney transplant recipients (KTRs), yet their molecular underpinnings remain poorly defined. We sought to synthesize current evidence on biomarker associations with muscle loss and function in the post renal transplant setting. **Methods:** A comprehensive search of PubMed/MEDLINE and Cochrane databases was conducted according to PRISMA guidelines. Studies evaluating biomarkers related to sarcopenia or sarcopenic obesity in adult and pediatric KTRs were included. Quality assessment was performed with the NHLBI tool. **Results:** Seven studies were included, encompassing 548 KTRs. Myostatin levels predicted sarcopenia in KTRs (cut-off: 390 pg/mL) and inversely correlated with Metabolic equivalent of Tasks (METs), handgrip strength (HGS), and graft performance. Although adiponectin was negatively correlated with body fat, its high-molecular-weight isoform was linked to lower muscle mass and long-term graft decline. Leptin was associated with sarcopenic obesity and lower estimated Glomerular Filtration Rate (eGFR). Insulin like Growth Factor-1 (IGF-1) independently predicted HGS but not muscle mass. Brain-derived neurotrophic factor (BDNF) levels predicted sarcopenia (cut off: 17.8 ng/mL) and reflected physical activity levels. Visfatin showed no association with sarcopenia but it was positively correlated with eGFR. Lastly, certain polymorphisms of Alpha-actinin-3 (ACTN3) were shown to genetically predispose to post-transplant sarcopenia. **Conclusions:** These emerging candidate biomarkers provide promising mechanistic insight into post-transplant muscle decline and may ultimately support more personalized risk assessment. Further validation is needed, and functional measures remain the most reliable clinical tools at present.

## 1. Introduction

Sarcopenia, defined as the progressive and generalized loss of skeletal muscle mass and function, has been identified as a major contributor to frailty, disability, and poor outcomes in patients with chronic kidney disease (CKD) [1,2]. Among kidney transplant recipients (KTRs), the prevalence of sarcopenia remains substantial (25–70%), despite improvements in renal function and overall survival [3]. The etiology of post-transplant sarcopenia is multifactorial, arising from a complex interplay of persistent low-grade inflammation, protein-energy wasting, insulin resistance, and disturbances in anabolic hormone signaling. Additional contributors include prolonged physical inactivity, extended exposure to corticosteroids and calcineurin inhibitors, and pre-existing muscle catabolism acquired during the dialysis period, all of which impair muscle regeneration and accelerate lean tissue loss after transplantation [3]. Sarcopenic obesity, defined as a condition in which excess adiposity coexists with muscle wasting, may further exacerbate functional impairment and graft-related complications [4,5]. Despite its clinical significance, there are no established molecular tools to reliably identify or predict sarcopenic disorders in this population.

In recent years, there has been a marked increase in research focusing on the identification of circulating biomarkers that may reflect or mediate changes in muscle metabolism, regeneration, and catabolism. Indeed, a wide array of myokines, adipokines, hormones, and cytokines have been implicated in muscle regulation in CKD patients both before and after transplantation [6,7]. These markers may provide mechanistic insights into sarcopenia and could potentially serve as adjuncts to imaging and functional testing in both diagnosis and longitudinal surveillance.

Nevertheless, existing literature on biomarkers of sarcopenia in KTRs is fragmented, with heterogeneous methodologies, variable definitions, and limited external validity. To date, no comprehensive synthesis has been conducted to consolidate biomarker data potentially associated with outcomes following renal transplantation. Bridging this gap is essential for understanding the molecular basis of muscle decline in kidney transplant recipients and for designing future interventions that encompass personalized rehabilitation, nutritional optimization, and risk-based immunosuppression [8]. The aim of this comprehensive review is to summarize and critically evaluate the available evidence on biomarkers potentially associated with sarcopenia and sarcopenic obesity in KTRs. Specifically, we explore molecular correlates of muscle mass, strength, physical activity, and graft function, with an emphasis on translational relevance and future research directions.

## 2. Materials and Methods

### 2.1. Study Design

The present comprehensive review was performed according to the Preferred Reporting Items for Systematic Reviews and Meta-Analyses (PRISMA) guidelines (end-of search date: 1 February 2025) [9]. The PECO criteria (Population/Participants, Exposure, Comparison and Outcome [10] were utilized to define our research question:

**P (Population)**: Adult and pediatric KTRs.

**E (Exposure)**: Presence of sarcopenia or sarcopenic obesity with biomarker profiling.

**C (Comparison)**: KTRs without sarcopenic features.

**O (Outcomes)**: The primary outcomes were graft survival, patient survival, and incidence of post-transplant complications. The secondary outcomes were perioperative and post-operative metrics including functional status, quality of life, and rehospitalization rates.

### 2.2. Inclusion and Exclusion Criteria

Eligible studies were English-language clinical investigations assessing biomarkers of sarcopenia or sarcopenic obesity in adult or pediatric (<18 years) renal transplant recipients, and designed as randomized controlled trials, or prospective non-randomized studies, or retrospective analyses.

The exclusion criteria were as follows: (i) non-English language publications, (ii) studies irrelevant to the research question, (iii) narrative and systematic reviews, meta-analyses, editorials, letters, commentaries, personal viewpoints, and errata, (iv) studies involving renal transplant recipients without evidence of sarcopenia or sarcopenic obesity, and (v) studies addressing sarcopenia or sarcopenic obesity in renal transplant recipients without reporting any biomarker assessment. In situations where multiple publications reported on overlapping patient populations, only the study with the largest cohort or the longest follow-up duration was selected for inclusion.

### 2.3. Search Strategy

A comprehensive literature search was performed independently by two independent investigators using the PubMed/MEDLINE and Cochrane CENTRAL databases. The search strategy employed Boolean operators and included the following terms: (renal transplant OR kidney transplant OR renal transplantation OR kidney transplantation) AND (sarcopenia OR sarcopenic obesity), with no restrictions on publication date. Discrepancies or disagreements regarding study eligibility were resolved through discussion with a third reviewer. Furthermore, reference lists of relevant articles were screened manually using the snowball approach to identify additional eligible studies [11]. The Rayyan reference and article manager software were utilized for all stages of the database search and study selection [12].

### 2.4. Data Extraction

A predefined set of criteria was established to guide the extraction of relevant data from the eligible studies. This process was conducted independently by two reviewers. All disagreements were resolved in consultation with a third reviewer.

The extracted data included: study identifiers (first author, year of publication, journal, institution, country, and PubMed ID), inclusion and exclusion criteria, demographics (sample size, age, sex), and clinical characteristics of kidney transplant recipients [including duration of dialysis, creatinine, estimated glomerular filtration rate (eGFR), blood glucose levels, total cholesterol, high density lipoprotein (HDL) cholesterol, triglycerides, serum albumin, hemoglobin]. Biomarker-related data were also recorded, including the name of the molecule, biological sample type (e.g., tissue or body fluid), measurement units, and analytical technique used. Captured anthropometric measures included body mass index (BMI), waist and hip circumference, percentage of body fat, lean body mass, skeletal muscle index (SMI), appendicular skeletal muscle index (ASMI), psoas muscle index (PMI), lean tissue index (LTI), smooth muscle index (sMI), intramuscular adipose tissue content (IMAC), and handgrip strength (HGS). Additionally, the level of physical activity, where reported, was recorded in metabolic equivalents (METs). Definitions for sarcopenia and sarcopenic obesity were documented, along with any available time-to-event outcomes following renal transplantation.

### 2.5. Descriptive Analysis

For continuous variables reported in at least two studies, we summarized the data as the median of the reported study-specific means, accompanied by the range of these means. For continuous data that were reported as median and interquartile (IQR) range, we used the Hozo et al. formula to estimate the respective mean and standard deviation (SD) [13]. When results were not offered in SD, but rather in confidence intervals (CI), we used Cochrane’s handbook equation for converting CI to SD [14]. Descriptive statistical analysis was performed using Stata/SE 18.0 software (Stata Corp, College Station, TX, USA).

### 2.6. Assessment of Study Quality

The methodological quality of the included studies was evaluated using the National Heart, Lung, and Blood Institute (NHLBI) assessment tool, which draws on established criteria from organizations such as the Agency for Healthcare Research and Quality, Cochrane Collaboration, U.S. Preventive Services Task Force, Scottish Intercollegiate Guidelines Network, and the NHS Centre for Reviews and Dissemination [15]. The NHLBI tool assigns scores from 1 to 14, with 0–5 indicating poor quality, 6–9 fair quality, and 10–14 good quality. Three independent reviewers assessed each study, and their evaluations were aggregated. The overall mean and standard deviation of the quality scores across all included studies were then calculated.

### 2.7. Protocol Registration

Our study was prospectively registered in the PROSPERO database (registration number: CRD42024583728).

## 3. Results

### 3.1. Study Selection

The initial database search identified 253 potentially relevant articles. Following title and abstract screening, 87 full-text articles were retrieved for comprehensive evaluation. After applying the predefined inclusion and exclusion criteria, seven studies met the eligibility requirements and were included in the final systematic review (Supplemental Figure S1). The manuscripts originated from a diverse range of institutions across Europe and Asia and were published between 2016 and 2024 (Table 1) [16,17,18,19,20,21,22]. Table 2 outlines the specific inclusion and exclusion criteria applied across the analyzed studies. Definitions of sarcopenia and sarcopenic obesity varied amongst evaluated manuscripts and can be explored in Table 3.

### 3.2. Quality Assessment

The mean NHLBI score of the review was 8.0 (SD: 1.4) and detailed quality assessment for each study is provided in Supplemental Table S1.

### 3.3. Kidney Transplant Recipient Characteristics

A total of 889 patients were included in this systematic review, of whom 548 (61.6%) were kidney transplant recipients. Laboratory data were available specifically for the transplant cohort (Table 4). Among these patients, the median duration of dialysis prior to transplantation was 34.2 [21.5–51.3] months, and the median age at transplantation was 44.3 [40.6–50.4] years. The proportion of male patients within the transplant group was 59.5%. Laboratory values in the transplant cohort at the time of assessment for sarcopenic disorders, revealed a median serum creatinine of 1.3 [1.1–1.6] mg/dL, eGFR of 64.3 [54.0–75.2] mL/min/1.73 m^2^, total cholesterol of 201.7 [189.0–214.4] mg/dL, HDL cholesterol of 46.8 [45.5–48.2] mg/dL, triglycerides of 154.4 [101.0–180.6] mg/dL, serum albumin of 4.2 [3.6–4.4] g/dL, and hemoglobin of 13.1 [12.9–13.9] g/dL. Details regarding immunosuppression regimens are summarized in Table 5.

### 3.4. Anthropometric Measurements in KTRs

Anthropometric and functional metrics used to diagnose sarcopenic disorders varied across the included studies (Table 6). BMI [16,17,18,19,21,22] and HGS [16,18,19,20,22] were each assessed in six and five studies, respectively. The median BMI among transplant recipients was 23.4 [20.4–26.3] kg/m^2^, and the median handgrip strength was 30.0 [26.9–33.1] kg (Table 7). The median body fat percentage was 33.1 [28.3–38.0] [16,22].

PMI was reported in two studies [17,21], while LTI—also evaluated in two studies—had median of 13.1 [11.9–14.2] kg/m^2^ [16,22]. On the other hand, SMI [18], ASMI [20], sMI [19], IMAC [17], lean body mass [16], as well as hip [16] and waist circumference [16] were measured in only one study each (Table 7).

**Table 6:** Captured biomarkers and anthropometric methods.

Study	Marker (Units)	Marker Biomaterial	Marker Measurment Method	Anthropometric Methods
**Czaja-Stolc et al., 2024 [22]**	Adiponectin (μg/mL), IL-6 (pg/mL), Leptin (ng/mL), Myostatin (pg/mL)	Blood serum	ELISA	BMI: (kg/m^2^), HGS (kg), LTI (kg/m^2^), Body fat (%)
**Fujimoto et al., 2023 [21]**	Alpha-actinin-3 (SNPs in ACTN3 gene)	Blood	RT-PCR	BMI (kg/m^2^), PMI (cm^2^/m^2^)
**Yasar et al., 2022 [20]**	Myostatin (ng/mL), IL-6 (pg/mL)	Blood serum	ELISA, Chemiluminescent Immunoassay	ASMI (kg/m^2^), HGS (kg)
**Koito et al., 2021 [18]**	BDNF (ng/mL), Myostatin (pg/mL)	Blood serum	ELISA	BMI (kg/m^2^), HGS (kg), MET (min/week), SMI (kg/m^2^)
**Yildirim et al., 2022 [19]**	IGF-1 (ng/mL)	Blood serum	Chemiluminescent Immunoassay	BMI (kg/m^2^), HGS (kg), sMI (smooth muscle mass/height^2^)
**Adachi et al., 2020 [17]**	Adiponectin (μg/mL)	Blood serum	ELISA	BMI (kg/m^2^), IMAC, PMI (cm^2^/m^2^)
**Małgorzewicz et al., 2016 [16]**	Adiponectin (mg/L), Leptin (μg/L), Visfatin (μg/L)	Blood serum	ELISA	Body fat (%), BMI (kg/m^2^), HGS (kg), Hip circumference (cm), Lean Body Mass (kg), LTI (kg/m^2^), Waist circumference (cm)

ASMI: Appendicular Skeletal Muscle Index, BDNF: Brain-Derived Neurotrophic Factor, BMI: Body Mass Index, ELISA: Enzyme-linked immunosorbent assay, HGS: Handgrip Strength, IGF-1: Insulin-like Growth Factor 1, IL-6: Interleukin 6, IMAC: Intramuscular Adipose Tissue Content, KTRs: Kidney Transplant Recipients, LTI: Lean Tissue Index, MET: Metabolic Equivalents, NA: Not Applicable, NR: Not reported, PMI: Psoas Mass Index, RT-PCR: Real Time Polymerase Chain Reaction, SD: Standard Deviation, SMI: Skeletal Mass Index, sMI: Smooth Muscle Index, SNPs: Single-Nucleotide Polymorphisms.

**Table 7:** Results of anthropometric studies.

Anthropometry/Study	KTRs	Hemodialysis	Peritoneal Dialysis	Non-Dialysis Dependent CKD	Control Group (No Kidney Disease)	*p*-Value
**ASMI**						
Yasar et al., 2022 [20] (kg/m^2^)	5.5 ± 1.9	5.4 ± 1.8	5.2 ± 1.5	5.0 ± 0.8	NA	0.709
**BMI**						
Czaja-Stolc et al., 2024 [22] (kg/m^2^)	26.2 ± 5.2	24.9 ± 3.9	26.7 ± 4.4	NA	NR	0.220
Fujimoto et al., 2023 [21] (kg/m^2^)	21.9 ± 11.0	NA	NA	NA	NA	NA
Koito et al., 2021 [18](kg/m^2^)	21.5 ± 2.8	NA	NA	NA	NA	NA
Yildirim et al., 2022 [19] (kg/m^2^)	26.3 ± 5.7	NR	NR	NR	25.6 ± 4.0	0.080
Adachi et al., 2020 [17] (kg/m^2^)	20.4 ± 2.8	NA	NA	NA	NA	NA
Małgorzewicz et al., 2016 [16] (kg/m^2^)	25.3 ± 4.2	NA	NA	NA	NA	NA
Mean value (kg/m^2^)	23.6 ± 5.3	24.9 ± 3.9	26.7 ± 4.4	NA	25.6 ± 4.0	
**Body fat**						
Czaja-Stolc et al., 2024 [22] (%)	38.0 ± 8.4	36.6 ± 10.8	34.1 ± 9.8	NA	NR	0.180
Małgorzewicz et al., 2016 [16] (%)	28.3 ± 8.4	NA	NA	NA	NA	NA
Mean value (%)	33.1 ± 8.4	36.6 ± 10.8	34.1 ± 9.8	NA	NA	
**HGS**						
Czaja-Stolc et al., 2024 [22] (kg)	33.1 ± 8.0	23.2 ± 10.5	28.6 ± 10.9	NA	NR	<0.001
Yasar et al., 2022 [20] (kg)	30.1 ± 16.8	25.4 ± 8.5	22.0 ± 8.7	24.8 ± 12.0	NA	0.025
Koito et al., 2021 [18](kg)	28.5 ± 7.1	NA	NA	NA	NA	NA
Yildirim et al., 2022 [19] (kg)	30.0 ± 11.5	NR	NR	NR	37.0 ± 13.2	<0.001
Małgorzewicz et al., 2016 [16] (kg)	26.9 ± 7.6	NA	NA	NA	NA	NA
Mean value (kg)	29.7 ± 10.2	24.3 ± 9.5	25.3 ± 9.8	24.8 ± 12.0	37.0 ± 13.2	
**IMAC**						
Adachi et al., 2020 [17]	-0.39	NA	NA	NA	NA	NA
**Lean Body Mass**						
Małgorzewicz et al., 2016 [16] (kg)	49.2 ± 11.9	NA	NA	NA	NA	NA
**LTI**						
Czaja-Stolc et al., 2024 [22] (kg/m^2^)	11.9 ± 2.0	11.6 ± 2.6	13.1 ± 2.4	NA	NR	0.007
Małgorzewicz et al., 2016 [16] (kg/m^2^)	14.2 ± 2.9	NA	NA	NA	NA	NA
Mean value (kg/m^2^)	13.0 ± 2.4	11.6 ± 2.6	13.1 ± 2.4	NA	NA	
**MET**						
Koito et al., 2021 [18] (min/week)	2021.5 ± 1737.7	NA	NA	NA	NA	NA
**PMI**						
Fujimoto et al., 2023 [21] (cm^2^/m^2^)	8.1 ± 7.6	NA	NA	NA	NA	NA
Adachi et al., 2020 [17]	3.4 (1 year after transplantation) 3.6 (5 years after transplantation)	NA	NA	NA	NA	NA
**SMI**						
Koito et al., 2021 [18] (kg/m^2^)	6.5 ± 0.8	NA	NA	NA	NA	NA
**sMI**						
Yildirim et al., 2022 [19] (kg/m^2^)	18.3 ± 2.1	NR	NR	NR	18.0 ± 2.5	0.074

ASMI: Appendicular Skeletal Muscle Index, BMI: Body Mass Index, CKD: Chronic Kidney Disease, HGS: Handgrip Strength, KTRs: Kidney Transplant Recipients, LTI: Lean Tissue Index, MET: Metabolic Equivalents, NA: Not Applicable, NR: Not reported, PMI: Psoas Mass Index, SMI: Skeletal Mass Index, sMI: Smooth Muscle Index.

### 3.5. Biomarkers in KTRs

Overall, myostatin [18,20,22] and adiponectin [16,17,22] were evaluated in three studies, respectively (Table 6). The median serum adiponectin level across studies was 5.4 [2.4–8.7] µg/mL, while the corresponding median myostatin level was not calculated due to significant heterogeneity in measurement techniques and units for this biomarker. Leptin was investigated in two studies, with a median serum concentration of 13.3 [9.3–17.3] ng/mL [16,22]. Interleukin-6 (IL-6), also examined in two manuscripts, had a median value of 3.8 [3.3–4.4] pg/mL, but was not correlated with sarcopenia in KTRs (Table 8) [20,22].

Insulin growth factor 1 (IGF-1) [19], alpha-actinin-3 [21], brain-derived neurotrophic factor (BDNF) [18], and visfatin [16] were only assessed in one study each (Table 8). Therefore, no summary statistics could be computed for these biomarkers.

**Table 8:** Results of biomarker analyses.

Biomarker/Study	KTRs	Hemodialysis	Peritoneal Dialysis	Non-Dialysis Dependent CKD	Control Group (No Kidney Disease)	*p*-Value
**Myostatin**						
Czaja-Stolc et al., 2024 [22] (pg/mL)	5558.6 ± 1771.9	3313.2 ± 1743.2	6391.8 ± 3088.4	NA	3683.0 ± 1460.3	<0.001 *
Yasar et al., 2022 [20] (ng/mL)	46.4 ± 21.6	378.0 ± 122.6	232.7 ± 97.6	318.8 ± 217.9	NA	<0.001
Koito et al., 2021 [18] (pg/mL)	314.5	NA	NA	NA	NA	NA
Mean value (pg/mL)	NA **	NA **	NA **	NA **	3683.0 ± 1460.3	
**Adiponectin**						
Czaja-Stolc et al., 2024 [22] (μg/mL)	2.4 ± 2.2	5.8 ± 5.0	8.1 ± 6.1	NA	2.7 ± 2.0	<0.001 *
Adachi et al., 2020 [17] (μg/mL)	5.2 ± 2.8 (1 year after transplantation) 5.6 ± 3.6 (5 years after transplantation)	NA	NA	NA	NA	NA
Małgorzewicz et al., 2016 [16] (mg/L)	8.7 ± 20.5	NA	NA	NA	NA	NA
Mean value (μg/mL)	5.5 ± 8.6	NA	8.1 ± 6.1	NA	2.7 ± 2.0	
**Leptin**						
Czaja-Stolc et al., 2024 [22] (ng/mL)	9.3 ± 10.9	10.4 ± 12.3	18.4 ± 21.6	NA	8.1 ± 10.7	0.040 *
Małgorzewicz et al., 2016 [16] (μg/L)	17.3 ± 20.5	NA	NA	NA	NA	NA
Mean value (ng/mL)	13.3 ± 15. 7	10.4 ± 12.3	18.4 ± 21.6	ΝA	8.1 ± 10.7	
**IGF-1**						
Yildirim et al., 2022 [19] (ng/mL)	206.5 ± 88.0	NR	NR	NR	169.7 ± 53.6	0.029
**BDNF**						
Koito et al., 2021 [18] (ng/mL)	16.8	NA	NA	NA	NA	NA
**Alpha-actinin-3 (SNPs in *ACTN3* gene)**						
Fujimoto et al., 2023 [21] (genotypes)	TT: 38.5% TC: 41.6% CC: 19.1%	NA	NA	NA	NA	NA
**Visfatin**						
Małgorzewicz et al., 2016 [16] (μg/L)	19.6 ± 4.7	NA	NA	NA	NA	NA
**IL-6**						
Czaja-Stolc et al., 2024 [22] (pg/mL)	3.3 ± 2.1	10.8 ± 9.3	7.6 ± 8.4	NA	1.4 ± 0.8	<0.001 *
Yasar et al., 2022 [20] (pg/mL)	4.4 ± 3.3	5.6 ± 3.2	6.7 ± 8.0	3.1 ± 3.3	NA	0.111
Mean value (pg/mL)	3.8 ± 2.7	8.2 ± 6.2	7.1 ± 8.2	3.1 ± 3.3	1.4 ± 0.8	

BDNF: Brain-derived Neurotrophic Factor, CKD: Chronic Kidney Disease, IGF-1: Insulin-like Growth Factor 1, IL-6: Interleukin 6, KTRs: Kidney Transplant Recipients, NA: Not Applicable, NR: Not reported, SNPs: Single-Nucleotide Polymorphisms * The control group was not included in the p-value estimation in the study by Czaja-Stolc et al., ** Summary statistics could not be performed due to significant heterogeneity in measurement techniques.

## 4. Discussion

The present systematic review consolidates current evidence on the molecular and clinical features of sarcopenia and sarcopenic obesity in renal transplantation. The included studies evaluated 548 KTRs with a median age of 44.3 years and a median dialysis duration of nearly three years prior to transplantation. This clinical profile likely suggests prolonged pre-transplant muscle loss and a reduced capacity for full post-transplant functional recovery.

Graft function appeared relatively preserved and nutritional markers such as serum albumin remained within stable ranges. However, metabolic parameters revealed subtle abnormalities. Median triglyceride levels were 154.4 mg/dL, total cholesterol reached 201.7 mg/dL, and BMI values (23.4 kg/m^2^) were clustered near the upper threshold of normal. These findings point toward persistent metabolic stress that may exacerbate muscular decline.

Functional assessments provided further insight. Of note, median handgrip strength was 30.0 kg, a value considered suboptimal given the predominantly male composition of the cohort [16]. Concurrently, the median body fat percentage was 31.1 percent, a finding consistent with increased adiposity [16]. This combination of diminished muscle strength and elevated fat mass supports the presence of sarcopenic obesity in our cohort.

### 4.1. Myostatin: Potential Associations

It should also be emphasized that the biomarker data presented in this review reveal a broad spectrum of molecular signatures with unique diagnostic utilities and relevance for targeted post-transplant monitoring and intervention strategies. As a starting point, we examined myostatin, a well-characterized myokine known to function as a potent inhibitor of skeletal muscle growth and maintenance. Myostatin binds to activin type II receptors and activates the SMAD (Mothers against decapentaplegic homolog) 2/3 signaling cascade, promoting expression of muscle degradation genes (atrogenes) while suppressing myogenic factors [23]. This action directly antagonizes the IGF-1 (insulin growth factor 1)/PI3K/Akt (Phosphatidylinositol 3-kinase/Protein kinase B) pathway, which promotes muscle protein synthesis [24].

Three studies evaluated the role of myostatin in sarcopenia following kidney transplantation [18,20,22]. First, Koito et al. found significantly higher serum myostatin levels in KTRs with reduced muscle mass compared to recipients with preserved muscle mass (362 pg/mL vs. 267 pg/mL, *p* = 0.024) [18]. Receiver operating characteristic (ROC) curve analysis yielded an area under the curve (AUC) of 0.69 for myostatin in identifying sarcopenia, with an optimal diagnostic cut-off value of 390 pg/mL. Importantly, a strong inverse correlation was observed between myostatin levels and METs in KTRs (r = −0.541, *p* < 0.001), suggesting that physical inactivity may contribute to elevated myostatin and subsequent muscle wasting [18].

In contrast, Yasar et al. reported the lowest serum myostatin levels in KTRs (46.4 ± 21.6 ng/mL) compared to patients on hemodialysis, peritoneal dialysis, or with non-dialysis-dependent CKD (378.0 ± 122.6 ng/mL, 232.8 ± 97.6 ng/mL, 318.8 ± 217.9 ng/mL, respectively; *p* < 0.001) [20]. Myostatin was also negatively correlated with handgrip strength (r = −0.203, *p* = 0.02) but did not correlate with ASMI (*p* = 0.35). Although only six KTRs were sarcopenic, myostatin independently predicted sarcopenia (OR 1.002, 95% CI: 1.001–1.005, *p* = 0.04) [20]. Interestingly, the authors hypothesized that immunosuppressive therapy may suppress myostatin production, potentially protecting against muscle loss. They also found a negative correlation between eGFR and myostatin levels (r = −0.626, *p* < 0.001), suggesting a link between graft function and muscle catabolism [20]. In summary, myostatin levels appear elevated in patients undergoing renal replacement therapy compared to those with non–dialysis-dependent CKD. Kidney transplantation tends to reduce myostatin levels and promote muscle mass recovery. Not-surprisingly, among transplant recipients, higher myostatin levels are associated with reduced muscle mass and sarcopenia.

### 4.2. Adiponectin: Potential Associations

Another relevant biomarker was adiponectin, an anti-inflammatory cytokine secreted by adipocytes and skeletal muscle, with well-established anti-atherogenic and insulin-sensitizing effects. Its levels typically decrease in obesity and rise with weight loss [25]. Mechanistically, adiponectin activates AMP (adenosine monophosphate)-activated protein kinase (AMPK), Sitruin-1 (SIRT1), and calcium-dependent pathways, which in turn stimulate PGC-1α (Peroxisome proliferator-activated receptor gamma coactivator 1-alpha), a key transcriptional coactivator involved in mitochondrial biogenesis and muscle metabolism [17].

Three studies explored the impact of adiponectin on muscle mass after renal transplantation [16,17,22]. In Małgorzewicz et al., mean adiponectin levels in KTRs were 8.7 ± 20.5 mg/L and negatively correlated with body fat (r = −0.18, *p* < 0.05), suggesting a protective metabolic profile against fat accumulation [16]. Adachi et al. investigated sarcopenia in 51 KTRs using psoas mass index and intramuscular adipose tissue content. PMI increased from 3.03 cm^2^/m^2^ pre-transplant to 3.36 at one year and 3.58 at five years. Interestingly, high molecular weight adiponectin correlated negatively with PMI at both one (r = −0.373, *p* = 0.007) and five years (r = −0.308, *p* = 0.028), and was independently associated with PMI reduction (β = −0.07, *p* = 0.003). These findings point to a paradoxical role of adiponectin, potentially contributing to muscle loss through effects on type II muscle fibers [17].

This apparent contradiction may reflect a compensatory upregulation of adiponectin in response to muscle wasting or metabolic stress. Alternatively, elevated levels might signal adipose tissue infiltration into muscle (myosteatosis), particularly of intermuscular fat, which secretes adiponectin locally. Furthermore, the differential roles of adiponectin isoforms—particularly the catabolic vs. anabolic signaling balance in type II muscle fibers—may contribute to sarcopenic phenotypes despite overall anti-inflammatory activity. Lastly, the observed correlation between low-molecular-weight adiponectin and eGFR changes (r_s_ = −0.362, *p* = 0.009) points to pleiotropic involvement in both renal and muscular remodeling [17]. Further elucidation of these mechanisms could clarify whether adiponectin serves as a biomarker of sarcopenia, a mediator, or both.

### 4.3. Leptin: Potential Associations

We subsequently assessed leptin, an adipose-derived hormone that regulates appetite and promotes inflammation via Janus kinase/signal transducers and activators of transcription (JAK-STAT), Phosphoinositide 3-kinase (PI3K), and Mitogen-activated protein kinase (MAPK) pathways [26]. It also contributes to muscle degradation via upregulation of tumor necrosis factor a (TNF-α), Interleukin-1 (IL-1), and Interleukin-6 (IL-6) [17].

The interplay of leptin with sarcopenic disorders in renal transplantation was evaluated by two studies [16,22]. In Małgorzewicz et al., 37.1% of the KTRs were overweight and 14.2% had obesity, with a mean leptin level of 17.3 ± 20.5 μg/L [16]. Leptin positively correlated with BMI and body fat (r = 0.5, *p* < 0.05) and negatively with lean mass (r = −0.5) and handgrip strength (r = −0.2, both *p* < 0.05), thereby supporting its role in sarcopenic obesity. Leptin also correlated negatively with eGFR (r = −0.3, *p* < 0.05), remaining significant in multivariate analysis, suggesting a link to graft dysfunction [16]. Similarly, Czaja-Stolc et al. reported significantly lower leptin levels in malnourished KTRs (7-Point Subjective Global Assessment < 5) compared to well-nourished individuals (8.8 ± 13.4 ng/mL vs. 13.3 ± 12.7 ng/mL; *p* = 0.04) [22]. Leptin also demonstrated the highest diagnostic accuracy for malnutrition among tested biomarkers (AUC = 0.69) [22].

### 4.4. Insulin Growth Factor 1: Potential Associations

Next, we sought to understand how IGF-1 affects the development of sarcopenic disorders in KTRs. IGF-1 is produced primarily by the liver and skeletal muscles, regulates growth hormone activity and promotes muscle protein synthesis via the PI3K/Akt/mTOR (mammalian target of rapamycin) and PI3K/Akt/GSK3β (Glycogen synthase kinase-3 beta) pathways [27,28]. IGF-1 also inhibits protein degradation by suppressing FoxO (Forkhead box O)-mediated ubiquitin ligases [28]. Yildirim et al. found significantly higher IGF-1 serum levels in 120 KTRs compared to 60 controls (206.5 ± 88.0 vs. 169.7 ± 53.6 ng/mL, *p* = 0.029) [19]. In this series, dynapenia was defined as HGS <26kg and <16 kg in men and women, respectively. In contrast, sarcopenia was defined as smooth muscle index (sMI)/BMI < 0.789 in men and <0.512 in women. Interestingly, IGF-1 was elevated in KTRs with normal muscle mass and handgrip strength (HGS) compared to those with dynapenia or sarcopenia (*p* = 0.037). Multivariate analysis also identified IGF-1 as an independent predictor of HGS (β = 2.314, *p* = 0.001), but not of muscle mass [19].

### 4.5. Brain-Derived Neurotrophic Factor: Potential Associations

BDNF is another cardinal mediator of sarcopenic disorders in KTRs. From a physiology standpoint, it is produced by both neurons and skeletal muscle, supports cognitive function, body mass regulation, and muscle regeneration via Tropomyosin receptor kinase B (TrkB) receptor–mediated pathways [29]. In Koito et al., KTRs with sarcopenia had significantly lower BDNF serum levels than their counterparts with normal muscle mass (15.7 ng/mL vs. 17.8 ng/mL, *p* = 0.013) [18]. Given that muscle contraction stimulates BDNF secretion, this reduction likely reflects diminished physical activity in sarcopenic patients. Notably, ROC analysis yielded an AUC of 0.712, with 17.8 ng/mL identified as the optimal BDNF threshold to diagnose sarcopenia. Furthermore, KTRs with low skeletal muscle index had significantly lower weekly METs than those with normal SMI (1504 ± 1183 vs. 2529 ± 2154 min/week, *p* = 0.001), and BDNF levels positively correlated with MET (r = 0.817, *p* < 0.001) [18]. These findings link physical activity to BDNF expression and underscore its potential utility in monitoring and guiding post-transplant rehabilitation.

### 4.6. Alpha-Actinin-3: Potential Associations

Alpha-actinin-3, encoded by the *ACTN3* gene, is a structural protein localized to Z-lines in skeletal muscle sarcomeres and is critical for muscle contraction. To date, limited data have correlated *ACTN3* with sarcopenic disorders in KTRs [30]. Most importantly, Fujimoto et al. investigated 65 KTRs and found that the CT and TT genotypes of the rs1815739 SNP (single nucleotide polymorphism) in *ACTN3* were significantly associated with decreased post-transplant psoas muscle index compared to the CC genotype (OR: 4.23, 95% CI: 0.05–0.97, *p* = 0.025) [21]. The T allele was more prevalent in KTRs with declining PMI (66.3% vs. 45.5%, OR: 2.34, *p* = 0.023). In contrast, delta PMI was significantly higher in CC genotype carriers compared to TT carriers (4.25 vs. −7.19, *p* = 0.023) [21]. Functionally, alpha-actinin-3 deficiency alters muscle metabolism, reducing glycogen phosphorylase activity and shifting fiber type composition toward slower, fatigue-resistant fibers, thereby compromising muscle strength. When combined with post-transplant immunosuppression, this genetic predisposition may accelerate sarcopenia in KTRs.

### 4.7. Visfatin: Potential Associations

Lastly, we explored the role of visfatin in the metabolomic and functional profile of KTRs. Visfatin is an adipokine secreted by intra-abdominal fat, with roles in adipogenesis and insulin-mimetic signaling [31]. Mechanistically, it promotes inflammation via IL-6, TNF-α, and IL-1β and may contribute to insulin resistance [31]. Małgorzewicz et al. reported a mean visfatin serum level of 19.6 ± 4.7 μg/L in KTRs, but found no association with BMI, body fat, or handgrip strength, suggesting limited utility as a sarcopenia marker [16]. Nevertheless, visfatin levels were positively correlated with eGFR (r = 0.3, *p* < 0.05), a finding confirmed on multivariate analysis, indicating potential value as a marker of renal graft function [16].

### 4.8. Strengths and Limitations

Methodological strengths of this review include (1) to our knowledge, the first comprehensive synthesis of biomarker associations with sarcopenia in kidney transplant recipients; (2) the use of a rigorous, systematic search strategy; (3) prior protocol registration in PROSPERO; and (4) standardized quality assessment across all included studies.

Nonetheless, several limitations should be acknowledged. First, only seven relatively small observational studies, stemming from three countries (Japan, Poland, Turkey), met inclusion criteria for data analysis. Second, the criteria used to define sarcopenia and sarcopenic obesity varied considerably across studies, and several did not quantify the exact number of renal transplant recipients affected by these conditions [16,17,18,21]. Third, while most studies focused on adult kidney transplant recipients, a few included both adult and pediatric populations without stratifying results by age. Similarly, the type of donor (living versus deceased) was inconsistently reported and not uniformly analyzed, potentially introducing clinical heterogeneity. Fourth, no data were available on key secondary outcomes such as quality of life or rehospitalization rates. Fifth, there was marked variability in the timing, methodology, and units of measurement for biomarker assessment, as well as inconsistent practices in biosample collection, storage, and calibration, which hindered comparability. Due to these multifaceted issues, a meaningful meta-analysis of anthropometric or biomarker outcomes was not feasible, and direct comparisons of individual biomarkers were limited by heterogeneity and data scarcity. Finally, although immunosuppressive therapy—particularly corticosteroids—has long been recognized as a major driver of post-transplant muscle catabolism, the included studies did not provide sufficient detail on the type, dose, duration, or cumulative exposure to these agents to permit stratified analyses. This limitation is notable given the differential mechanistic effects of commonly used immunosuppressants on muscle and adipose biology. Corticosteroids accelerate muscle atrophy through activation of the ubiquitin–proteasome pathway, whereas calcineurin inhibitors (CNIs) promote myofibrosis and impair mitochondrial function—each of which could meaningfully alter circulating biomarker profiles and confound associations with sarcopenia. The absence of regimen-specific reporting limits interpretability and may partially underlie the heterogeneity observed across studies. Future biomarker investigations should systematically account for immunosuppression—particularly cumulative steroid exposure and CNI dosing—in order to more accurately delineate true biological relationships with muscle mass and function.

### 4.9. Future Directions

Future research should focus on validating these biomarker associations in larger, multicenter prospective cohorts of kidney transplant recipients. Integrating molecular profiling with functional and imaging-based assessments may offer prognostication benefits. Interventional studies targeting modifiable pathways, such as myostatin or adiponectin signaling, are warranted. Genetic screening, including *ACTN3* variants, could inform personalized rehabilitation strategies. Ultimately, translating biomarker insights into clinical tools may improve long-term musculoskeletal and graft outcomes as well as overall quality of life.

## 5. Conclusions

This review highlights key biomarkers that could be potentially associated with sarcopenic disorders in kidney transplant recipients. Myostatin appears to be elevated in sarcopenic KTRs and inversely correlated with METs, handgrip strength, and eGFR. Adiponectin seems to demonstrate both protective metabolic effects and a paradoxical association with muscle loss and impaired graft function. Leptin levels have been correlated with sarcopenic obesity, reduced muscle function, and impaired graft performance. IGF-1 independently predicts handgrip strength but not muscle mass, emphasizing its relevance to muscle function over mass. BDNF levels seem to be reduced in sarcopenic patients and strongly associated with physical activity. Limited data suggest that *ACTN3* polymorphisms may be associated with post-transplant muscle loss, indicating a genetic predisposition to sarcopenia. Visfatin does not appear to correlate with sarcopenic disorders but may positively track with eGFR, supporting its role in graft monitoring. Nevertheless, functional assessments remain the most reliable and validated clinical tools in this population. At this stage, molecular biomarkers may offer valuable mechanistic insight into muscle wasting processes and guide hypothesis generation, but further prospective validation is essential before their incorporation into biomarker-guided screening or therapeutic stratification.

## Figures and Tables

**Table 1:** Included studies.

Study	Center	Country	Total Population
**Czaja-Stolc et al., 2024 [22]**	University Clinical Center of Gdansk	Poland	180
**Fujimoto et al., 2023 [21]**	Kobe University Hospital, Kobe	Japan	65
**Yasar et al., 2022 [20]**	Gazi University, Faculty of Medicine, Department of Nephrology, Ankara	Turkey	130
**Koito et al., 2021 [18]**	Kansai Medical University Hospital, Osaka	Japan	40
**Yildirim et al., 2022 [19]**	Nephrology Clinic of Baskent University Hospital, Ankara	Turkey	240
**Adachi et al., 2020 [17]**	Department of Nephrology, Kanazawa Medical University School of Medicine, Daigaku, Uchinada, Ishikawa	Japan	51
**Małgorzewicz et al., 2016 [16]**	Department of Nephrology, Transplantology and Internal Disease, Medical University of Gdansk	Poland	183

**Table 2:** Study inclusion and exclusion criteria.

Study	Inclusion Criteria	Exclusion Criteria
**Czaja-Stolc et al., 2024 [22]**	KTRs aged ≥18 years with a minimum of 3 months since kidney transplantation, in stable clinical condition (no recent surgical or infectious complications related to the transplant, including signs of allograft rejection). HD and PD patients aged ≥18 with a minimum dialysis duration of 3 months and who provided written informed consent to participate.	Inability to provide informed consent, cognitive impairment, or active oncological disease.
**Fujimoto et al., 2023 [21]**	KTRs	Graft loss unrelated to immunologic causes, death unrelated to transplantation and insufficient DNA obtained from blood samples.
**Yasar et al., 2022 [20]**	Patients aged 18–65 years with CKD in a clinically stable condition, under regular follow-up in the nephrology department between March 2018 and May 2019.	Patients with limb amputation, wheelchair use, immobility, active infection, a history of surgery within the last 3 months, thyroid or liver dysfunction, degenerative neurological or psychiatric disease, active malignancy, pregnancy or lactation, or a history of hospitalization within the last 3 months.
**Koito et al., 2021 [18]**	Patients aged over 20 years, who had undergone renal transplantation at least 6 months prior to the study period, and with an eGFR > 30 mL/min/1.73 m^2^	KTRs with communication disability, coexisting malignancy, arthritis or neuromuscular disorders affecting both extremities, congestive heart failure or nephrotic syndrome, implantation of a pacemaker or prosthesis, or severe arrhythmogenic risk.
**Yildirim et al., 2022 [19]**	KTRs aged 18–65 years, who had received a kidney transplant at least 6 months prior to enrollment and had eGFR > 30 mL/min/1.73 m^2^. CKD patients with a similar age, sex, and BMI profile to KTRs and eGFR < 30 mL/min/1.73 m^2^ for at least 6 months. Healthy individuals with a similar age, sex, and BMI profile to KTRs, eGFR > 60 mL/min/1.73 m^2^ and no known history of kidney disease.	Individuals with active or prior malignancy, use of more than 10 mg of prednisolone (or equivalent) within the last 6 months, arthritis or neuromuscular disease, congestive heart failure, severe edema, presence of a pacemaker or prosthetic valve or severe electrolyte disturbances.
**Adachi et al., 2020 [17]**	KTRs who had undergone kidney transplantation since 1998 and had maintained stable renal function for at least 6 months thereafter.	Vulnerable populations, including prisoners, individuals with reduced mental capacity due to illness or advanced age, and children.
**Małgorzewicz et al., 2016 [16]**	KTRs in the early post-transplantation period (30 to 180 days).	NR

BMI: Body Mass Index, CKD: Chronic Kidney Disease, DNA: Deoxyribonucleic acid, eGFR: estimated Glomerular Filtration Rate, HD: Hemodialysis, KTRs: Kidney Transplant Recipients, NR: Not Reported, PD: Peritoneal Dialysis.

**Table 3:** Definitions of sarcopenia and sarcopenic obesity.

Study	Definition of Sarcopenia	Definition of Sarcopenic Obesity
**Czaja-Stolc et al., 2024 [22]**	HGS < 27 kg for men and <16 kg for women and LTI < 14 kg/m^2^	BMI ≥ 30 kg/m^2^ and HGS < 27 kg for men and <16 kg for women and LTI < 14 kg/m^2^
**Fujimoto et al., 2023 [21]**	PMI calculated from CT-derived cross-sectional areas of the bilateral psoas muscles at the L4/5 level, normalized to height (cm^2^/m^2^)	NR
**Yasar et al., 2022 [20]**	ASMI < 7 kg/m^2^ in men and <5.5 kg/m^2^ in women and HGS < 27 kg in men and <16 kg in women/Decreased ASMI identified as probable sarcopenia	NR
**Koito et al., 2021 [18]**	SMI < 7 kg/m^2^ for men and <5.4 kg/m^2^ for women	NR
**Yildirim et al., 2022 [19]**	SMI/BMI < 0.789 in men and <0.512 in women	NR
**Adachi et al., 2020 [17]**	Loss of the mass, strength and skeletal muscles	Obese patients with sarcopenia
**Małgorzewicz et al., 2016 [16]**	NR	Skeletal muscle wasting and dysfunction associated with pathological accumulation of adipose tissue

ASMI: Appendicular Skeletal Muscle Index, BMI: Body Mass Index, HGS: Handgrip Strength, LTI: Lean Tissue Index, NR: Not reported, PMI: psoas muscle mass index, SMI: Skeletal Mass Index.

**Table 4:** Kidney transplant recipient characteristics.

Study	Czaja-Stolc et al., 2024 [22]	Fujimoto et al., 2023 [21]	Yasar et al., 2022 [20]	Koito et al., 2021 [18]	Yildirim et al., 2022 [19]	Adachi et al., 2020 [17]	Małgorzewicz et al., 2016 [16]	Mean Value
**Number of KTRs**	52	65	37	40	120	51	184	
**Age at transplantation**	50.4 ± 11.6	44.3 ± 13.9	NR	NR	NR	40.6 ± 17.5	NR	45.1 ± 14.3
**Duration of CKD (months)**	21.5 ± 19.8	46.8 ± 57.6	13.4 ± 10.4	21.6 ± 48.0	NR	52.3 ± 76.7	NR	31.1 ± 42.5
**Sex% (Male/Female)**	55.8/44.2	63.1/36.9	60.0/40.0	72.5/27.5	50.0/50.0	60.8/39.2	54.3/45.7	59.5/40.5
**Creatinine (mg/dL)**	1.4 ± 0.6	1.2 ± 0.4	1.1 ± 0.4	1.3 ± 0.4	1.2 ± 0.4	NR	1.6 ± 0.9	1.3 ± 0.5
**eGFR (mL/min/1.73 m^2^)**	54.0 ± 23.2	NR	75.2 ± 27.0	NR	74.4 ± 23.7	NR	54.1 ± 25.6	64.4 ± 24.9
**Blood glucose (mg/dL)**	NR	NR	92.2 ± 19.3	NR	NR	NR	NR	92.2 ± 19.3
**Total cholesterol (mg/dL)**	NR	NR	NR	189.0 ± 50.4	NR	NR	214.4 ± 50.5	201.7 ± 50.4
**HDL** **(mg/dL)**	NR	NR	NR	NR	45.5 ± 13.2	NR	48.2 ± 14.0	46.8 ± 13.6
**Triglycerides (mg/dL)**	NR	NR	133.7 ± 69.4	101.0 ± 58.6	180.6 ± 98.5	NR	175.1 ± 76.1	147.6 ± 75.6
**Serum Albumin (g/dL)**	4.1 ± 0.3	NR	4.3 ± 0.3	4.4 ± 0.4	4.2 ± 0.5	NR	3.6 ± 0.5	4.1 ± 0.4
**Hemoglobin (g/dL)**	13.9 ± 1.7	NR	NR	13.0 ± 2.0	13.1 ± 1.9	NR	12.9 ± 1.6	13.2 ± 1.8

CKD: Chronic Kidney Disease, eGFR: estimated Glomerular Filtration Rate, HDL: High-Density Lipoprotein, KTRs: Kidney Transplant Recipients, NA: Not Applicable, NR: Not reported.

**Table 5:** Immunosuppression regimens.

Study	Immunosuppression Strategy
**Czaja-Stolc et al., 2024 [22]**	Glucocorticosteroids Calcineurin inhibitors Antimetabolites (Mycophenolate mofetil)
**Fujimoto et al., 2023 [21]**	Corticosteroids Calcineurin inhibitors mTOR inhibitor
**Yasar et al., 2022 [20]**	Glucocorticosteroids (prednisolone) Calcineurin inhibitor (tacrolimus/cyclosporine) Antimetabolite (Mycophenolate mofetil/mycophenolic acid) mTOR inhibitors (everolimus)
**Koito et al., 2021 [18]**	Glucocorticosteroids (prednisolone) Calcineurin inhibitor (tacrolimus) Antimetabolite (Mycophenolate mofetil) anti-CD25 (basiliximab)
**Yildirim et al., 2022 [19]**	Glucocorticosteroids (prednisolone) Calcineurin inhibitor (tacrolimus) Antimetabolite (Mycophenolate mofetil) mTOR inhibitors (sirolimus)
**Adachi et al., 2020 [17]**	Steroids Calcineurin inhibitors Antimetabloites
**Małgorzewicz et al., 2016 [16]**	NR

CD: cluster differentiation; mTOR (mechanistic target of rapamycin); NR: Not reported.

## Data Availability

Raw data may be provided by the authors upon request.

## Supplementary Materials

The following supporting information can be downloaded at: https://www.mdpi.com/article/10.3390/jcm14248943/s1, Figure S1. PRISMA flow diagram. Table S1. NHLBI Quality Assessment Scale for Observational Cohort and Cross-Sectional Studies.
