# Supplementary material for: Biomarkers of Sarcopenia and Sarcopenic Obesity in Renal Transplant Recipients: A Systematic Review and Evidence Quality Assessment
Source: J Clin Med. 2025 Dec 18;14(24):8943. doi: 10.3390/jcm14248943 (PMC12734315; doi:10.3390/jcm14248943)
Supplement: Supplementary file 1 [file jcm-14-08943-s001.zip › jcm-4035377-supplementary.pdf]

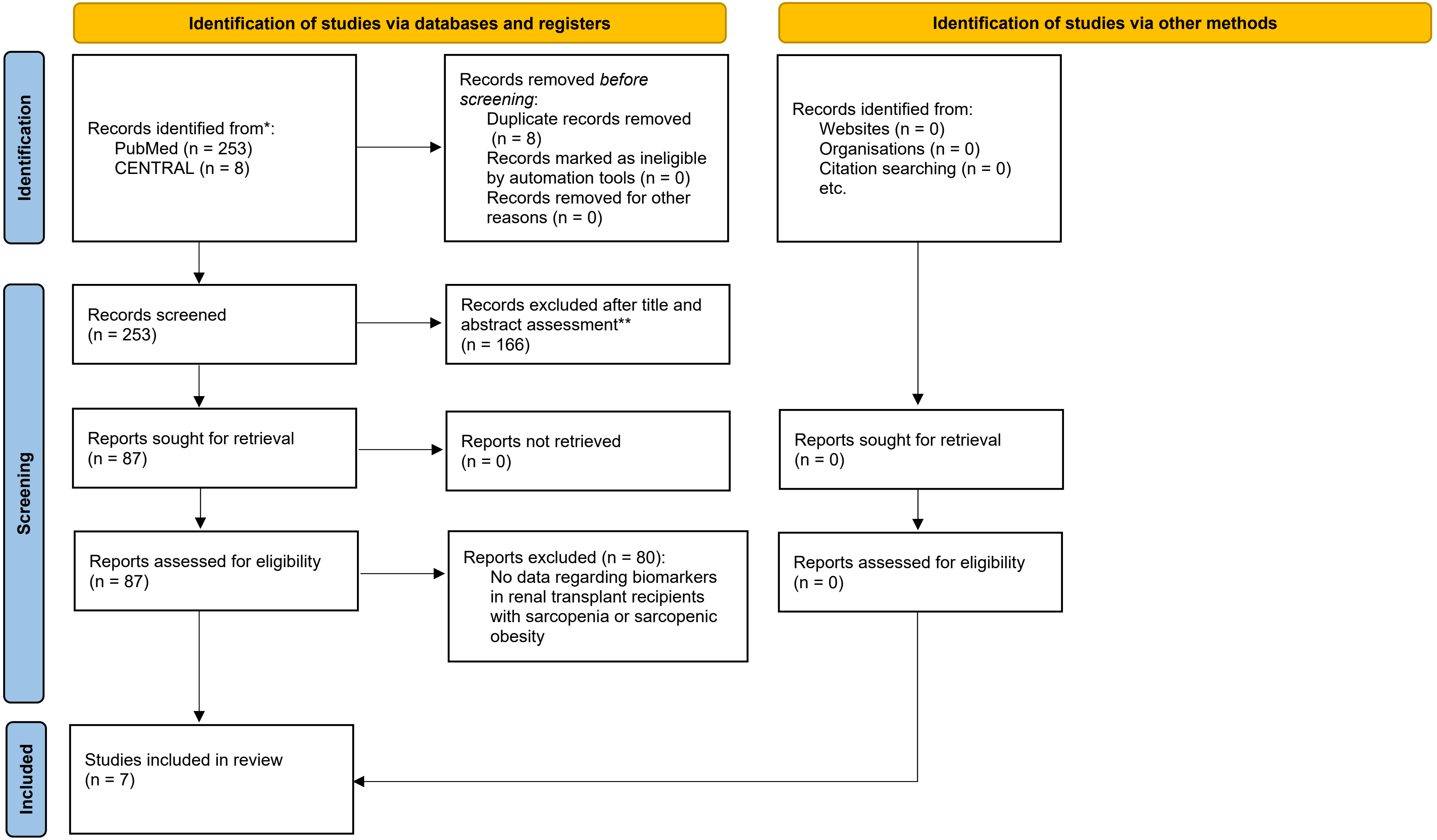

From: Page MJ, McKenzie JE, Bossuyt PM, Boutron I, Hoffmann TC, Mulrow CD, et al. The PRISMA 2020 statement: an updated guideline for reporting systematic reviews. BMJ 2021;372:n71. doi: 10.1136/bmj.n71. For more information, visit: http://www.prisma-statement.org/

Supplemental Figure S1. PRISMA flow diagram

Supplemental Table S1. NHLBI Quality Assessment Scale for Observational Cohort and Cross-Sectional Studies

PMID	39125361	37037724	35122995	34253381	34716090	32612097	27496430
**First author**	Czaja-Stolc	Fujimoto	Yasar	Koito	Yildirim	Adachi	Małgorzewicz
**Was the research question or objective in this paper clearly stated?**	1	1	1	1	1	1	1
**Was the study population clearly specified and defined?**	1	1	1	1	0	1	1
**Was the participation rate of eligible persons at least 50%?**	1	1	1	1	1	1	1
**Were all the subjects selected or recruited from the same or similar populations (including the same time period)? Were inclusion and exclusion criteria for being in the study prespecified and applied uniformly to all participants?**	0	1	1	1	1	1	1

**Was a sample size justification, power description, or variance and effect estimates provided?**	0	0	0	0	0	0	0
**For the analyses in this paper, were the exposure(s) of interest measured prior to the outcome(s) being measured?**	0	0	0	0	0	1	0
**Was the timeframe sufficient so that one could reasonably expect to see an association between exposure and outcome if it existed?**	0	1	0	0	0	1	0

**For exposures that can vary in amount or level, did the study examine different levels of the exposure as related to the outcome (e.g., categories of exposure, or exposure measured as continuous variable)?**	1	1	1	1	1	1	1
**Were the exposure measures (independent variables) clearly defined, valid, reliable, and implemented consistently across all study participants?**	1	1	1	1	1	1	1
**Was the exposure(s) assessed more than once over time?**	0	1	0	0	0	1	0
**Were the outcome measures (dependent variables) clearly defined, valid, reliable, and implemented consistently across all study participants?**	1	1	1	1	1	1	1

**Were the outcome assessors blinded to the exposure status of participants?**	0	0	0	0	0	0	0
**Was loss to follow-up after baseline 20% or less?**	NA	NA	NA	NA	NA	NA	NA
**Were key potential confounding variables measured and adjusted statistically for their impact on the relationship between exposure(s) and outcome(s)?**	1	0	1	0	1	1	0
**Total**	7	9	8	7	7	11	7
**Quality rating**	Fair	Fair	Fair	Fair	Fair	Good	Fair

The NHLBI scale ranges from 1 – 14; with a score of 0-5 denoting poor quality, 6-9 fair quality and 10-14 suggesting good quality.
NHLBI: National Heart, Lung, and Blood Institute; N/A: not applicable
